# Effects of vatinoxan in rats sedated with a combination of medetomidine, midazolam and fentanyl

**DOI:** 10.1186/s13028-024-00744-y

**Published:** 2024-05-31

**Authors:** Emily Lindh, Anna Meller, Marja Raekallio

**Affiliations:** 1 Department of Equine and Small Animal Medicine, Faculty of Veterinary Medicine, University of Helsinki, Koetilantie 4, P.O.Box 57, Helsinki, FI-00014 Finland; 2 Laboratory Animal Center, Mustialankatu 1 G, P.O.Box 29, Helsinki, FI-00014 Finland

**Keywords:** Alpha2-adrenoceptor agonist, Alpha2-adrenoceptor antagonist, Diuresis, Hyperglycaemia, Medetomidine, Subcutaneous

## Abstract

**Background:**

Alpha2-adrenoceptor agonists (α_2_-agonists) are widely used in animals as sedatives and for pre-anaesthetic medication. Medetomidine has often been given subcutaneously (SC) to rats, although its absorption rate is slow and the individual variation in serum drug concentrations is high via this route. In addition, α_2_-agonists have various effects on metabolic and endocrine functions such as hypoinsulinaemia, hyperglycaemia and diuresis. Vatinoxan is a peripherally acting α_2_-adrenoceptor antagonist that, as a hydrophilic molecule, does not cross the blood-brain barrier in significant quantities and thus alleviates peripheral cardiovascular effects and adverse metabolic effects of α_2_-agonists. Aim of this study was to evaluate the effects of vatinoxan on sedation, blood glucose concentration, voiding and heart and respiratory rates and arterial oxygen saturation in rats sedated with subcutaneous medetomidine, midazolam and fentanyl.

**Results:**

Onset of sedation and loss of righting reflex occurred significantly faster with vatinoxan [5.35 ± 1.08 (mean ± SD) *versus* 12.97 ± 6.18 min and 6.53 ± 2.18 *versus* 14.47 ± 7.28 min, respectively]. No significant differences were detected in heart and respiratory rates and arterial oxygen saturation between treatments. Blood glucose concentration (18.3 ± 3.6 *versus* 11.8 ± 1.2 mmol/L) and spontaneous urinary voiding [35.9 (15.1–41.6), range (median) *versus* 0.9 (0–8.0) mL /kg/min] were significantly higher without vatinoxan.

**Conclusions:**

Acceleration of induction of sedation, alleviation of hyperglycaemia and prevention of profuse diuresis by vatinoxan may be beneficial when sedating rats for clinical and experimental purposes with subcutaneous medetomidine, midazolam and fentanyl.

## Background

Alpha2-adrenoceptor agonists (α_2_-agonists), such as detomidine, medetomidine, dexmedetomidine, xylazine and romifidine, are widely used in animals as sedatives and for preanaesthestic medication. They induce sedation by binding to α_2_-adrenoceptors in the central nervous system and inhibiting the release of noradrenaline from nerve endings [1]. Medetomidine is often administered subcutaneously (SC) to rats and it is widely used in laboratory rodent anaesthesia [2], although its absorption rate is slow and the individual variation in serum drug concentration is high via this route [3].

The use of α_2_-agonists is associated with well known adverse effects. They stimulate peripheral adrenoceptors, which are present in several tissues, such as the liver, pancreas, kidneys, adipose tissue, eye [4], vascular walls and platelets [5]. Activity in the vasculature causes vasoconstriction and increased arterial blood pressure, followed by a reflex bradycardia [6]. In addition, they have various effects on metabolic and endocrine functions. For example, α2-agonists reduce insulin release from storage vesicles in pancreatic β-cells, leading to hypoinsulinaemia and hyperglycaemia [7]. They also increase diuresis and natriuresis by centrally reducing the production of antidiuretic hormone (ADH) [8], by blocking the peripheral effects of ADH in renal tubules [9] and by increasing the concentration of natriuretic peptide in plasma [10]. Despite these potential adverse effects of medetomidine, the combination of medetomidine, midazolam and fentanyl has been suggested to provide reliable sedation in rats [11–13]. However, it has also been recognised that anaesthesia procedures may have profound effects on plasma metabolite and hormone concentrations in experimental rats [14].

Vatinoxan (previously MK-467 or L659,066) is a peripherally acting α_2_-adrenoceptor antagonist that, as a hydrophilic molecule, does not cross the blood-brain barrier in significant quantities [15]. Mitigation of peripheral cardiovascular effects have been described in many animal species [16–18] when administering vatinoxan together with various α_2_-agonists. Vatinoxan also alleviates the adverse metabolic effects of α_2_-agonists [19–21]. However, when administered intravenously (IV), it does not markedly affect sedation induced by α_2_-agonists [22, 23].

Previous studies in various animal species have focused on IV and intramuscular (IM) routes of administration of the α_2_-agonist-vatinoxan combination. In dogs, alleviation of the early cardiovascular effects of α_2_-agonists by vatinoxan depends on the route of administration [21, 24]. Intravenous dosing bypasses the absorption phase, and an immediate dose-dependent alleviation or prevention of α_2_-agonist induced adverse cardiovascular events by vatinoxan has been noted [16]. When administered IM, an early increase in peripheral resistance and mean blood pressure caused by α_2_-agonists occurs, probably due to the slower absorption rate of vatinoxan than medetomidine [21]. Intramuscularly, a medetomidine-vatinoxan combination led to a faster onset and shorter duration of sedation than medetomidine alone, likely because of the hastened absorption of medetomidine by vatinoxan [21]. The SC administration route of the combination has not been previously reported in animals.

The aim of this study was to investigate the effects of vatinoxan on sedation, blood glucose concentration, urination, heart and respiratory rates and arterial oxygen saturation (SpO_2_) in rats sedated with medetomidine, midazolam and fentanyl. The hypothesis was that vatinoxan administration with medetomidine, midazolam and fentanyl would result in a faster onset of sedation and reduced cardiovascular and metabolic adverse effects compared to the same drug combination without vatinoxan.

## Methods

### Animals

Ten male and two female adult Wistar rats, weighing 256–506 g were used. The rats were laboratory animals residing in controlled environments. They were paired and accommodated within the animal facilities at the Laboratory Animal Center of the University of Helsinki, housed in ventilated cages where temperature, humidity, and light-dark cycles were maintained. The rats were provided with a standard rodent diet (Teklad 8460 standard rodent diet, Harlan Teklad) and had access to purified water ad libitum. The colonies from which these animals were selected are regularly monitored for disease and the colonies are free of any notifiable or communicable disease. They had no external signs of disease or injury at the time of the study. The rats were surplus animals used for teaching purposes and gathered for the study and used by the 3R principle of reduction, for concurrent education of caretakers on anaesthesia and monitoring of laboratory animals. The Regional State Administrative Agency for Southern Finland approved the study (ESAVI/21,803/2020). The licence fulfils the requirements of the European Union legislation and the Animal Research: Reporting of In Vivo Experiments guidelines (ARRIVE).

### Study design

Rats were divided into six pairs, matched for sex, age and weight. One rat in each pair was randomly selected, by drawing lots, to be sedated with SC medetomidine hydrochloride (HCl) (0.25 mg/kg, Domitor vet; 1 mg/mL; Orion Pharma, Finland) midazolam HCl (3.0 mg/kg, Midazolam Hameln; 5 mg/mL; Hameln Pharma Gmbh, Germany) and fentanyl citrate (0.01 mg /kg, Fentanyl Hameln; 0.05 mg/mL; Hameln Pharma Gmbh, Germany) (MMF), and the other rat was administered a commercial mixture of medetomidine 0.25 mg/kg and vatinoxan 5.0 mg/kg hydrochlorides (Zenalpha; medetomidine HCl 0.5 mg/mL + vatinoxan HCl 10 mg/mL; Vetcare Ltd, Finland) in the same syringe with midazolam and fentanyl (MMFV). The drugs were mixed within two hours before administration and the initial volume was 1 ml/kg in both groups. The rats were spontaneously breathing room air and body temperature was actively maintained using infrared heating pads (Far Infrared Surgical Warming Pad DCT-25; Kent Scientific Corporation; CT; USA). The pair of rats was sedated at the same time and they were laying on the same heat pad on dorsal recumbency and observed by the same caretakers blinded for the treatments.

### Monitoring

The onset of sedation and the loss of the righting reflex were timed. Sedation was identified as when voluntary movements disappeared, thereafter righting reflex was tested by placing the rat into dorsal recumbency. The righting reflex was considered absent when the rat stayed on its back, after which it was laid on the heating pad. If the rat were to return to sternal recumbency, testing was repeated after approximately 1 min.

Assessments were performed at 10, 20 and 30 min after drug injection. The depth of sedation was monitored with the presence of the pelvic limb nociceptive withdrawal reflex, where the skin between the toes of the pelvic limb was pinched by fingernails while the leg was extended. Heart rate and SpO_2_ were measured with a pulse oximeter (NONIN PalmSAT 2500 VET; Nonin Medical Inc; MN; USA). The sensor was placed on the hind paw immediately below the foot pad. Rectal temperature was measured by thermometer (Microtherma 2 Type T Thermometer, RET-3-ISO Animal rectal probe for mice; Agntho’s; Sweden). Respiratory rate was monitored visually by counting the number of breaths for 15 s. Skin colour was evaluated by comparing the skin between the paw pads of the front paws with a paint colour model (Teknos Prague Valentine T1540) representing the normal colour of rat skin. Compared against the colour model, the skin was considered to be either pale (1), normal (2) or hyperaemic (3).

A drop of blood (0.3 µL) was obtained by puncturing the tail vein with a 20 G needle, and blood glucose concentration was measured with a glucometer (AlphaTrak 2, Abbott Laboratories; IL; USA), with the setting Rat CRL/CD, at 30 min after drug injection. According to the user manual [25], 1.9 mmol/L was subtracted from the measured values.

Spontaneously voided urine was collected with an absorbent pad. The pad was weighed, placed under the rear end of the rat after the righting reflex had disappeared, and weighed again when it was removed 30 min after injection. Urine output (mL/kg/h) was calculated by dividing the volume of urine voided (mL) by rats’ body weight (kg) and divided by the time spent on the heating pad (h).

At the end of the experiment, rats were humanely euthanised by cervical dislocation under anaesthesia. Before cervical dislocation, an appropriate level of sedation was confirmed by administrating 80–100 mg/kg pentobarbital (Mebunat vet.; sodium pentobarbital 60 mg/mL; Orion, Finland) diluted 1:1 with lidocaine (Lidor vet.; lidocaine hydrochloride 20 mg/mL; VetViva Richter GmbH, Austria) intraperitoneally when needed.

### Statistical analysis

Experimental sample size (6 pairs) was calculated for achieving a power of 80% and a level of significance of 5% (two sided), for detecting a mean of the differences of 5 mmol/L between pairs, assuming the standard deviation (SD) of the differences to be 3 mmol/L in blood glucose concentration. For detecting a mean of differences of 10 min between pairs, assuming the SD of the differences to be 5 min in the onset of sedation, with a power of 80% and a level of significance of 5% (two sided), 5 pairs were needed.

The normal distribution of data was tested with the Shapiro-Wilk test (IBM SPSS Statistics 28, IBM Corp., NY; USA). For the normally distributed data, repeated measures ANOVA was performed after which post hoc t-test was used and for other data Wilcoxon signed rank test was used for pairwise comparisons between treatments, as appropriate. Normally distributed data are stated as mean and standard deviation (SD) and other data as median (minimum-maximum). Values of *P* < 0.05 were considered significant.

## Results

Onset of sedation and loss of righting reflex occurred significantly faster with MMFV than with MMF (Table 1). Limb withdrawal reflex was present during the entire postinjection period in three rats in the MMF group and in one rat in the MMFV group. Ten minutes after MMF injection, three rats were still alert, not allowing heart and respiratory rates, SpO_2_ and rectal temperature to be measured, and therefore, they are not reported in Table 2. None of the rats had righting reflex ten minutes after MMFV, and thus, all measurements could also be performed for them at that time point. No significant differences were detected in heart and respiratory rates, SpO2 and rectal temperature between treatments, but skin colour was paler with MMF than with MMFV (Table 2). Blood glucose concentration and the amount of spontaneous urinary voiding were significantly higher with MMF than with MMFV (Table 1). The highest blood glucose concentration measured in any rat was 24.3 mmol/L after MMF and 13.8 mmol/L after MMFV.

**Table 1:** Onset of sedation, loss of righting reflex, blood glucose concentration and spontaneous urinary voiding

	MMF	MMFV	*P*-value
Onset (minutes)	12.97 ± 6.18	5.35 ± 1.08	0.028
Loss of righting (minutes)	14.47 ± 7.28	6.53 ± 2.18	0.029
Glucose (mmol/L)	18.3 ± 3.6	11.8 ± 1.2	0.007
Urination (mL/kg/ h)	35.9 (15.1–41.6)	0.9 (0–8.0)	0.028

Onset of sedation, loss of righting reflex, blood glucose concentration (mean ± SD) and calculated amount of spontaneous urinary voiding (median, range) in rats sedated with subcutaneous medetomidine, midazolam and fentanyl without (MMF) and with vatinoxan (MMFV), and the P-value for significance between treatments

**Table 2:** Heart and respiratory rates, SpO_2_, rectal temperature and skin colour score

		10 min	20 min	30 min
Heart rate (1/min)	MMF	NA	264 ± 11	244 ± 13
	MMFV	318 ± 23	278 ± 16	255 ± 29
SpO_2_ (%)	MMF	NA	87 ± 6	86 ± 5
	MMFV	90 ± 2	90 ± 2	88 ± 3
Respiratory rate (1/min)	MMF	NA	68 ± 14	67 ± 14
	MMFV	67 ± 17	59 ± 9	53 ± 11
Rectal temperature (^O^C)	MMF	NA	36.7 ± 1.0	35.9 ± 0.7
	MMFV	36.8 ± 0.6	36.7 ± 0.5	36.4 ± 0.5
Skin colour score^#^	MMF	2 (1–2)	2 (1–2)	2 (1–2)
	MMFV	2 (2–3)	2.5 (2–3)*	2.5 (2–3)*

Heart and respiratory rates, arterial oxygen saturation (SpO_2_), rectal temperature (mean ± SD) and skin colour score (median, range) in rats sedated with subcutaneous medetomidine, midazolam and fentanyl (MMF) without (*N* = 6) and with (*N* = 6) vatinoxan (MMFV). * significant difference between treatments (*p* < 0.05)

^#^ 1 = pale, 2 = normal, 3 = hyperaemic

NA, not assessed, because three rats were not sedated at 10 min

## Discussion

Vatinoxan hastened the onset of sedation, alleviated hyperglycaemia and prevented diuresis in rats sedated with SC medetomidine, midazolam and fentanyl. No significant differences were detected in heart and respiratory rates, SpO_2_ or rectal temperature between treatments. The results from the female rats were interpreted as consistent with those from the male rats, taking into account their respective ranges. Therefore, the data from the females were managed together with the males.

The prolonged onset of sedation observed in our study after MMF was probably caused by slow absorption of medetomidine by the SC route [3]. Vatinoxan significantly hastened the onset of sedation in rats. In the MMF group, it took more than twice as long as in the MMFV group to achieve loss of righting reflex. Previous studies with dogs have suggested that vatinoxan can locally attenuate medetomidine-induced vasoconstriction at the site of IM injection, thus accelerating the absorption of drugs administered in the same syringe [21, 26]. Therefore, it is probable that the faster onset of sedation observed in our study with MMFV than with MMF was caused by accelerated absorption of SC administered medetomidine, midazolam and fentanyl by vatinoxan. The quick onset of the sedative effect with vatinoxan via the SC route is beneficial from the clinical point of view since it may increase the feasibility of the SC route in rats. Their muscle mass is small, and SC injection causes less pain than IM injection, the latter of which is generally not recommended for small rodents [2].

Bradycardia was noted after MMF compared to the reference values for awake rats (250–450 beats per minute) [2], and vatinoxan was not able to alleviate it significantly. Fentanyl itself is known to induce bradycardia [27] and in an earlier study, dexmedetomidine did not worsen the cardiovascular depression associated with high-dose opiates in rats [28]. Although our dose was smaller than in those studies, it is possible that the bradycardia detected in our study was partly mediated by the activation of opioid receptors, which vatinoxan could not antagonise. However, the skin colour was less pale with vatinoxan than without it, suggesting a reduction of medetomidine-induced vasoconstriction by vatinoxan. This finding agrees with an earlier study, where the medetomidine-induced reduction of superficial circulation and its improvement by vatinoxan were illustrated by thermographic imaging of dog footpads [29].

Vatinoxan significantly attenuated the effect of medetomidine on blood glucose concentration in rats. The upper reference value for postprandial blood glucose concentration in rats is considered to be 10.4 mmol/L [30]. In the MMF group, glucose values ​​of more than double the rat’s physiological postprandial value were observed. In the rats receiving vatinoxan, blood glucose concentration also tended to be slightly higher than the physiological values. In horses, a similar effect has been reported with the detomidine/vatinoxan combination, which caused the blood glucose concentration to increase but significantly less and for a shorter period of time than in those receiving detomidine alone [20]. Stability in blood glucose is clinically beneficial in reliably monitoring rats’ well-being [31] and differentiating from stress-induced hyperglycaemia or other disease processes. Moreover, stability in blood glucose may be of interest in experimental glucose-sensitive measurement and imaging protocols [32].

Vatinoxan prevented medetomidine-induced diuresis in rats. The median volume of spontaneously voided urine of rats receiving MMF was more than 15 times the physiological urinary excretion of rats, 2.3 mL/kg/h (5.5 mL/100 g/day) [33]. Measuring the volume of spontaneous urinary voiding may have underestimated the urinary excretion, if the excreted volume after MMFV treatment was small enough to be held in the bladder while the rat was placed on the absorbent pad. However, this possible bias was minor compared to the substantial volumes voided after MMF. The average water consumption of a rat is 100 mL/kg/d [33]. In the MMF group, the calculated spontaneous urinary voiding was up to approximately 40 mL/kg/h. Considering that diuresis has been reported to last up to 4 h in dogs [34] and 3 h in horses [35] sedated with α2-agonists, it can be assumed that blocking the diuretic effect with vatinoxan is beneficial for maintaining the animal’s fluid balance and preventing dehydration. In the kidneys, glucose is normally absorbed back into the bloodstream in the proximal tubules, but when the blood glucose concentration exceeds 10 mmol/L in dogs and 16.6 mmol/L in cats, the kidney threshold is exceeded and glucose is excreted with the urine, causing osmotic diuresis [36]. On the other hand, medetomidine 20 and 40 µg/kg IV in dogs did not increase blood glucose above the renal threshold, although significantly increased diuresis was seen at both doses [37]. A study in rats, however, reported glucosuria with blood glucose concentration of 26 ± 2.8 mmol/L and 36 ± 4.5 mmol/L in healthy and diabetic rats, respectively [38]. Therefore, although glucose concentration in urine was not measured in our study, it is unlikely that glucosuria would have been the main cause of the increased diuresis observed in the MMF group. More probably, it was induced by central inhibition of antidiuretic hormone (ADH) secretion [8] and by various peripheral mechanisms such as inhibiting the renin-angiotensin-aldosterone system and reducing ADH facilitation in the renal tubules [39]. Furthermore, activation of α_2_-receptors in the musculature of the bladder and urethra may have caused spontaneous leakage of urine [40]. However, urine leakage alone cannot explain the enormous volume of urine voided after MMF treatment. Since vatinoxan does not remove central effects, it can be considered that the effect of medetomidine on urine output is mainly peripherally regulated.

We were unable to measure the basal metabolic and vital values of awake individual animals. In addition, we did not evaluate the recovery from sedation, as the experiment was terminal, and the rats were euthanised under anaesthesia. It would have been beneficial to gather information on the basal values of the group, especially individual rats’ metabolic status since there may be some variation in rat strains. Also, further investigation is needed on the effects of vatinoxan on the overall duration of sedation in rats sedated with medetomidine. Furthermore, the group size was small, but matching the rats to allow the use of paired tests increased statistical power, and we were able to detect significant differences between groups in our primary outcomes, i.e. onset of sedation, blood glucose concentration and volume of urinary voiding. Further studies with larger sample sizes are needed to investigate cardiovascular effects including the effects on blood pressure.

## Conclusions

The accelerated onset of sedation and mitigation of physiological disruptions, such as the attenuation of severe hyperglycaemia and the prevention of excessive diuresis, represent potential beneficial outcomes of vatinoxan in the sedation of rats for clinical or experimental purposes using subcutaneous administration of medetomidine, midazolam, and fentanyl.

## Data Availability

The datasets used and/or analysed during the current study are available from the corresponding author on reasonable request.

## Declarations

### Prior publication

Data have not been published previously.
